# Augmented Versus Virtual Reality in Education: An Exploratory Study Examining Science Knowledge Retention When Using Augmented Reality/Virtual Reality Mobile Applications

**DOI:** 10.1089/cyber.2018.0150

**Published:** 2019-02-13

**Authors:** Kuo-Ting Huang, Christopher Ball, Jessica Francis, Rabindra Ratan, Josephine Boumis, Joseph Fordham

**Affiliations:** ^1^ Department of Journalism, Center for Emerging Media Design and Development, Ball State University, Muncie, Indiana.; ^2^ Department of Media and Information, Michigan State University, East Lansing, Michigan.; ^3^ Clinical and Translational Science Institute, University of Rochester, Rochester, New York.; ^4^ Department of Communication, Michigan State University, East Lansing, Michigan.

**Keywords:** augmented reality, virtual reality, immersion and presence, science education

## Abstract

The propagation of augmented reality (AR) and virtual reality (VR) applications that leverage smartphone technology has increased along with the ubiquity of smartphone adoption. Although AR and VR technologies have been widely utilized in the educational domain, there remains a dearth of empirical research examining the differences in educational impact across AR and VR technologies. The purpose of our exploratory study was to address this gap in the literature by comparing AR and VR technologies with regard to their impact on learning outcomes, such as retention of science information. Specifically, we use a two-condition (AR vs. VR) between-subjects' design to test college students' science-knowledge retention in response to both auditory and visual information presented on a Samsung S4 smartphone app. Our results (*N* = 109) suggest that VR is more immersive and engaging through the mechanism of spatial presence. However, AR seems to be a more effective medium for conveying auditory information through the pathway of spatial presence, possibly because of increased cognitive demands associated with immersive experiences. Thus, an important implication for design is that educational content should be integrated into visual modalities when the experience will be consumed in VR, but into auditory modalities when it will be consumed in AR.

## Introduction

With the increasing popularity and adoption of smartphones, we have seen a subsequent rise in augmented reality (AR) and virtual reality (VR) applications that leverage smartphone technology. Low-cost consumer VR headsets such as the Samsung Gear VR, Google Cardboard, and Google Daydream have made digital realities more accessible than ever before.^1^ Moreover, the use of AR through mobile devices has shown new educational possibilities and implications for different populations in various contexts.^2^ As a result, smartphone-based AR and VR technologies and applications have been drawing the attention of researchers and educators alike. VR and AR have been implemented in educational settings for years and empirical evidence demonstrates that both technologies help improve student's learning outcomes and enjoyment in different contexts and across various subjects.^3,4^

AR and VR have been widely applied to teaching, learning, and instructional design. However, there is currently a lack of research that empirically compares the educational effects of AR and VR technologies. AR is a technology that blends digital information with the information from physical-world environments, enabling users to interact with virtual objects and view the physical environment (usually through a digital camera in a mobile phone or tablet) simultaneously.^5^ In contrast, VR involves real-time immersive simulations completely through digital graphics.^6^ Therefore, AR integrates virtual objects into a physical space, whereas VR blocks out information from the physical environment that subsequently transports users to a fully virtual world. In other words, VR provides users with a feeling of being psychologically immersed in a virtual environment,^7^ and AR allows users to interact with both virtual items and objects in the real world. Consequently, the affordances of AR and VR are fundamentally different.

To our knowledge, no studies have compared the impacts of smartphone-based AR and VR technologies in the context of information retention. Comparisons of AR and VR have tended to focus on visual/graphical aspects of these technologies with little if any attention to auditory information. This exploratory study aims to fill this gap in the literature by empirically comparing AR and VR technologies with regard to their effects on science-learning outcomes. Specifically, this experiment compares the effects of AR and VR technologies on college students' retention of science knowledge presented through auditory and visual information. The goal of this research was to provide insights into the possible psychological and theoretical mechanisms with a focus on the role of presence that has been used to account for the impacts of educational technologies on learning outcomes (e.g., Wood and Cifuentes^8^ and Ibáñez et al.^9^). Furthermore, this study sheds light on how to best implement these emerging technologies in educational settings.

This study aims to answer two questions. First, what are some of the possible psychological and cognitive mechanisms that might explain any potential differences between AR and VR in an educational context? Second, is AR or VR a more effective tool/medium for educating students about science? To answer these questions, this study compares the effects of AR and VR on learning outcomes in the context of solar system education. To better understand the underlying theoretical mechanisms, this study utilizes a model of spatial presence experiences and perception load theory. The literature surrounding spatial presence and the perceptual load of attention may help uncover the psychological and cognitive differences and affordances of AR and VR technology that may have a subsequent impact on retaining science information.

### The role of presence

VR technology-based instruction may improve students' learning outcomes in various subjects and contexts,^4^ in part because such technologies facilitate spatial presence (or just presence), that is, the subjective experience of physically being in a virtual or mediated environment.^10^ When media users allocate their attention to spatial information from the mediated environment, they form a mental representation of the environment.^11^ After they accept the mental representation and treat the virtual environment as a physical world, they begin to experience the sense of presence that has been positively associated with enjoyment of media use.^12^ In contrast, AR provides users with additional digital information integrated into existing physical environments.^13^ Because AR technologies focus on the physical environment and do not transport users to an artificial world like VR,^14^ AR technologies are less likely to elicit feelings of presence. Based on this reasoning, we hypothesize:

**H1: Individuals who experience a VR environment—compared to an AR environment with the same digital content—will report a) higher levels of attention to the mediated environment, b) presence, and c) enjoyment.**

The enhanced spatial representation, as well as representational fidelity (i.e., mostly visual information) in VR, can potentially improve learning outcomes.^4^ However, digital instructional technologies often involve visual and auditory information and thereby force learners to divide their attention between different modalities simultaneously. Perceptual load theory suggests that attentional capacity is shared across vision and hearing. Therefore, the high perceptual load of attention during a visual task may lead to a lower level of sensitivity in auditory detection because of divided attention, which is regarded as load-induced deafness.^15^ In this sense, the high perceptual load of attention during an immersive visual experience in VR may lead to a higher level of presence that may overload perceptual (i.e., attentional) resources, thereby limiting individuals' ability to retain auditory-related information. Therefore, we propose the following hypotheses:

**H2: Individuals who consume visual and auditory content in VR compared to AR will a) exhibit greater retention of visual information but b) lower retention of auditory information.**

**H3: Differences between VR and AR for a) visual and b) auditory learning outcomes will be mediated by the level of presence such that more presence will enhance visual outcomes and hinder auditory outcomes.**

## Methods

### Participants

To test the proposed hypotheses, this study used a two-condition (AR vs. VR) between-subjects design with participants randomly assigned to each condition. A total of 109 participants (57 in the AR condition and 52 in the VR condition) were recruited from an interdepartmental research subject pool at a large university in the United States. In exchange for participation in the institutional review board-approved study, participants received a small amount of extra credit (∼1 percent of their final grade). Almost 75 percent of the participants were women (*n* = 81, 74.3 percent) and the average age was 20.5 years old (standard deviation [*SD*] = 1.61). There were no significant differences in gender, age, and race between participants across the conditions.

### Materials

**FIG. 1.**
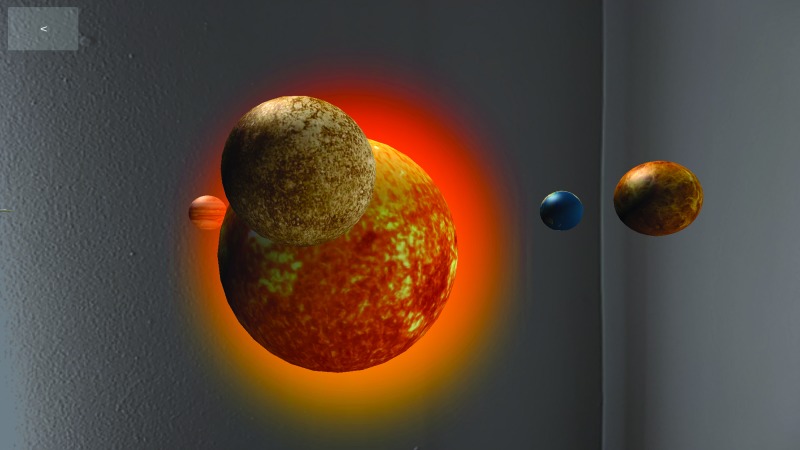
Screenshots of solar system—space museum in the AR mode. AR, augmented reality. Color images are available online.

**FIG. 2.**
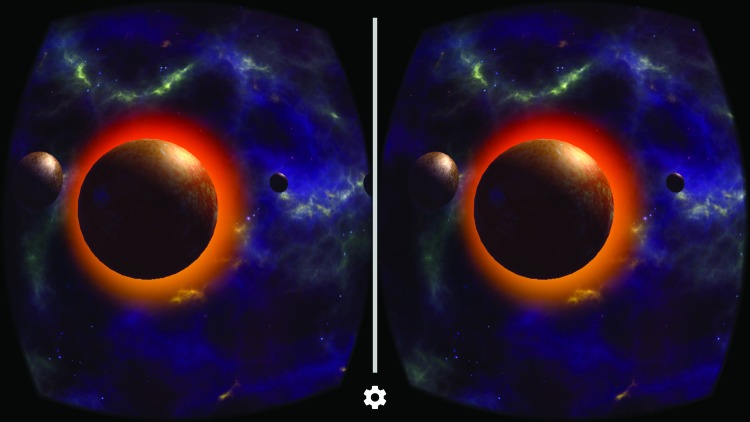
Screenshots of solar system—space museum in the VR mode. VR, virtual reality. Color images are available online.

To test our hypotheses, the study used a mobile app, Solar System—Space Museum, on a Samsung S4 smartphone to display the same digital content (space-related visual and auditory information) in either AR or VR mode (Figs. 1 and 2). The information presented in the app included both three-dimensional visual representations of the solar system and planets and auditory commentary about astronomy, planets, and the solar system. The app itself was extremely easy to use, requiring the participants to observe the scale representations of the solar system and planets while listening to the auditory commentary. The auditory information played automatically upon startup of the experience and did not require any specific actions from the participants. The menu navigation was different when using the AR or VR modes within the app. Specifically, users needed to tap the screen to navigate menus in AR while they used gazed selection to navigate the same menus in VR. To minimize the difference of menu navigation between conditions, the researchers navigated the menus for the participants in both conditions.

**FIG. 3.**
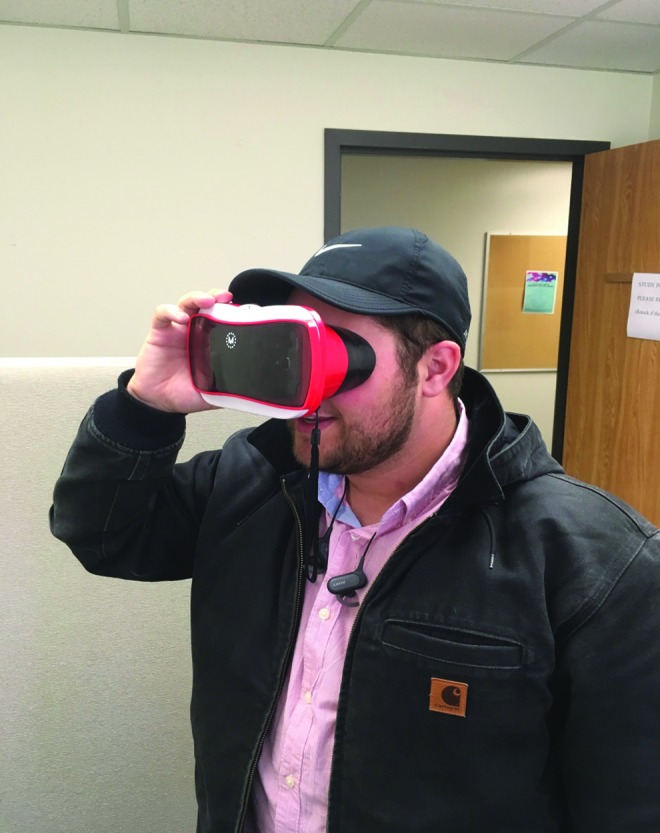
The experimenter demonstrated how to use the VR headset to view the digital content. The only difference between AR and VR conditions is viewing the digital content with or without the headset. Color images are available online.

To maintain consistency in participants' experiences across both conditions, participants were required to hold the mobile device in their hands while using the app (i.e., looking around the solar system). In the AR mode, participants held the smartphone in front of their faces and viewed the digital content displayed as a noninteractive layer on top of an image of the physical environment fed from the phone's front-facing camera. In the VR mode, participants held a Mattel VR Viewmaster mobile phone-based VR headset to their eyes and viewed the digital content displayed in front of a white background (Fig. 3). In both modes, the user could turn the device 360° to look around at the content. The auditory content was identical between modes. The auditory information itself was transmitted through the built-in speakers of the smartphone. The study was conducted in a 10′ × 8′ segmented office space with an empty desk, beige walls, and beige curtains without and distracting objects or noise.

### Procedure

Upon arrival at the laboratory, participants were first asked to complete a pretest questionnaire with questions measuring their science (i.e., solar system) knowledge. As part of a larger study, participants were randomly presented with one of two articles related to gender disparity and digital technologies, one that said women and men are equally skilled in their use of technologies and another that said men outperform women. This manipulation was not found to have an effect on this study's outcome variables and thus is not reported in our results. A future article will analyze whether the manipulation influenced belongingness and acceptance of gender stereotypes in STEM fields.

Afterward, participants were instructed on how to use the AR or VR application (depending on condition). Participants then received visual and auditory information from the mobile app for 5 minutes. This was enough time for each participant to hear and visually see all the solar system content presented in the application. Participants then answered a posttest questionnaire with questions about their psychological and cognitive feelings of spatial presence and a new set of science (i.e., solar system) knowledge questions.

### Measures

Attention to the mediated environment was measured with an existing scale (α = 0.93)^16^ that assesses the level of attention allocation to a mediated environment (e.g., “I dedicated myself completely to the game”). Spatial presence was measured with a revised scale on immersive virtual technologies (α = 0.90)^17^ (e.g., “How much did the virtual world seem like the real world?”). Enjoyment was also measured using an existing scale (α = 0.91)^18^ (e.g., “the informational environment is enjoyable”). For all measures, participants were asked to indicate their agreement on a seven-point scale (1 = strongly disagree; 7 = strongly agree). The survey items for each scale were summed and averaged to create overall variable scores.

Participants' science knowledge was assessed with multiple-choice questions developed specifically for this study. Previous research has implemented similar instruments to evaluate students' learning outcomes after exposure to AR^19^ or VR^20^ environments. The pretest contained 10 questions related to general knowledge taken from basic information provided in the app (e.g., “Is Earth larger or smaller than most of the other planets”). The posttest contained 10 questions regarding planets and our solar system. Of these questions, five were derived from the auditory information presented in the mobile app (e.g., “Which are considered the gas giants?”) and five from the visual information presented (e.g., “What color was the planet that seemed to get the closest to you?”). To ensure that participants' preexisting knowledge did not influence the results of the experiment, knowledge at the pretest was compared. There was no significant difference in the score between VR (*M* = 5.59, *SD* = 1.55) and AR (*M* = 5.58, *SD* = 1.66) conditions (Table 1).

**Table 1.** Mean, Standard Deviations, and *t*-Tests of Outcome Variables (*N* = 109)

	*Virtual reality (*n* = 52)*		*Augmented reality (*n* = 57)*		t-*Test (df = 107)*	
*Variables*	M	SD	M	SD	t	p*-Value*
Attention	6.11	0.78	5.78	0.90	2.04	0.043^*^
Presence	4.33	1.16	3.23	1.25	4.79	0.000^***^
Enjoyment	3.79	0.84	3.48	0.90	1.88	0.063
Science knowledge (pretest)	5.59	1.55	5.58	1.66	1.51	0.135
Auditory knowledge (posttest)	6.51	3.05	7.71	2.49	2.28	0.024^*^
Visual knowledge (posttest)	3.89	2.13	3.05	2.13	2.07	0.041^*^

SD, standard deviation.

^*^ *p* < 0.05; ^***^p < 0.001.

## Results

To test the first hypothesis, a series of independent samples *t*-tests were conducted (Table 1). Results of the *t*-tests indicated that participants in the VR condition paid more attention to the mediated environment than those in the AR condition. Therefore, H1a was supported. Results also indicated that those in the VR condition reported higher levels of spatial presence compared with those in the AR condition. Therefore, H1b was also supported. Finally, participants in the VR condition reported greater levels of enjoyment than the participants in the AR condition. Therefore, H1c was also supported. In other words, the first hypothesis was completely supported by our findings.

To test the second hypothesis another set of independent samples *t*-tests were conducted (Table 1). Results revealed that participants in the VR condition scored higher on the visual information retention compared with the AR condition. Therefore, H2a was supported. Furthermore, participants in the AR condition had higher scores on auditory information retention, supporting H2b. In other words, the second hypothesis was completely supported by our findings.

**FIG. 4.**
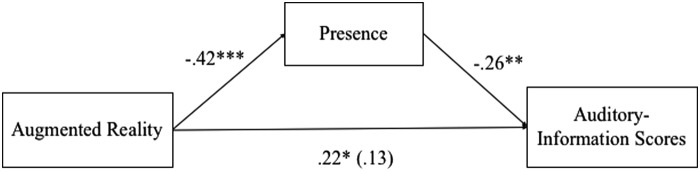
Standardized regression coefficients for the relationship between AR and auditory information scores as mediated by presence. *p < 0.05; **p < 0.01; ***p < 0.001.

Finally, to test the third hypothesis, a mediation test was conducted using the PROCESS path-analysis macro (Model 4). The results showed that being in the VR and AR conditions influenced participants' feelings of spatial presence differently, which later impacted their science-learning outcomes differently. Specifically, the relationship between AR and VR and participants' scores on auditory-related information was mediated by spatial presence at a significant level, *t*(106) = −2.06, *p* < 0.05, 95 percent confidence interval (−0.88 to −0.02). In other words, participants in the VR condition reported higher levels of spatial presence that accounted for the lower scores on auditory information. However, interestingly, the impact of VR on participants' scores on visual information retention was not mediated by spatial presence. Therefore, the third hypothesis was partially supported. For the results of the mediation analysis see Figure 4.

## Discussion

This study compared the impacts of AR and VR on knowledge retention when using a mobile phone platform. Participants in the VR condition allocated more attention to the mediated environment, perceived higher feelings of spatial presence, and reported more enjoyment than those in the AR condition. In other words, being in the VR condition resulted in more psychological and cognitive responses to the media compared with the AR condition. Furthermore, those psychological and cognitive responses to the media helped participants retain more knowledge that was presented visually in the mobile app.

However, although participants in the AR condition retained less visual-related science information, they retained more auditory-related science information compared with those in the VR condition. Participants' feelings of spatial presence played a mediating role in the relationship between modality and science learning. To be more specific, when participants were in the VR condition, they paid more visual attention to the mediated environment so they received more information of the same modality (i.e., visual) and less in the different modality (i.e., auditory). This finding is consistent with previous research on shared attentional capacity across visual and auditory perception.^15^ In contrast, participants in the AR condition had weaker cognitive and psychological responses (i.e., attention, presence, and enjoyment) to the virtual environment, allowing them to dedicate more cognitive and attentional resources toward retaining the auditory information. The level of spatial presence explained the impacts of VR and AR technologies on participants' scores on auditory information, but not on visual information. This unexpected finding indicates the need for more research on the relationship between spatial presence and learning across these two media modalities.

This study has important theoretical implications for the literature on AR and VR, namely, that these two modalities place different cognitive demands on users. In particular, VR seems to be more immersive and engaging through the psychological mechanism of spatial presence. However, the cognitive demands of such immersive experiences may make AR a more effective medium for conveying auditory (or other nonvisual) information. Researchers should continue to explore the psychological and cognitive differences between these two media modalities.

These findings also have important implications for educators and designers/developers of interactive media and technologies. For educators who wish to emphasize content that is communicated visually, VR (compared with AR) seems to be a better technology because it draws attention to the visual information presented in the environment. However, if the digital learning technologies include important information conveyed through auditory channels, AR seems to be more effective because it frees up attentional/cognitive resources otherwise dedicated to visual channels, allowing the auditory information to be processed more thoroughly. Designers of VR and AR applications for educational purposes should consider the cognitive demands and modalities that are best suited for specific types of content. When designing VR experiences, it may be best to embed relevant information visually into the environment. Likewise, when designing AR experiences, it may be best to communicate relevant information in an auditory format.

### Limitations and future research

This study has various limitations. The study utilized a mostly female, college-age sample. Future researchers should expand the generalizability of their findings by expanding the representativeness of population samples used. The study only included self-reported questions when measuring participants' feelings of spatial presence. Future research should incorporate nondisruptive *in situ* measures (e.g., secondary reaction-time tasks) to reliably confirm influence on participants' feelings of presence. This study primarily focused on attentional resources and did not directly measure the participants' cognitive load, and the measure of attention does not necessary reflect their perceptual load despite the link between the two variables.^15^ Future research should further examine the role of cognitive or perceptual load while using these two technologies and how cognitive or perceptual load influences participants' feelings and presence as well as learning outcomes. The auditory information presented in the application was arguably less concrete (e.g., planet facts) than the visually presented information (e.g., planet sizes), so future research should control for information concreteness as a potential confound between modalities. The study did not control for participants' previous experience using AR and VR technologies that were likely novel for many participants. Future research should investigate whether previous experience using these technologies might have an impact on the efficacy of AR and VR for education. Furthermore, future research should explore whether the consistency of auditory and video information (i.e., irrelevant vs. relevant) or types of auditory outputs (e.g., headphone vs. speaker of the phone) influence or even optimize learning outcomes across modes.

## Conclusion

Cost-effective and portable AR and VR technologies provided by smartphone-based mobile applications provide tremendous potential for education. The ability to transport students to the stars with VR or to create a scale solar system on students' desks with AR has the potential to change the way students interact with science information. This study has shown that AR and VR can both be used effectively to teach science-based information. However, AR and VR have their own set of strengths and weaknesses that should be considered while integrating these technologies into learning environments. In the end, both technologies provide students with an exciting new educational reality.
